# Cardiac Manifestations in a Group of Romanian Patients with Gaucher Disease Type 1 (a Monocentric Study)

**DOI:** 10.3390/diagnostics11060989

**Published:** 2021-05-29

**Authors:** Cecilia Lazea, Simona Bucerzan, Camelia Al-Khzouz, Anca Zimmermann, Ștefan Cristian Vesa, Ioana Nașcu, Victoria Creț, Mirela Crișan, Carmen Asăvoaie, Diana Miclea, Paula Grigorescu-Sido

**Affiliations:** 1 1st Pediatric Discipline, Mother and Child Department, “Iuliu Hațieganu” University of Medicine and Pharmacy, Clinic Pediatrics I, Emergency Pediatric Hospital, 400370 Cluj-Napoca, Romania; bucerzansimona@yahoo.com (S.B.); alkhzouz@yahoo.com (C.A.-K.); 2 Department of Genetic Diseases, Emergency Pediatric Hospital, “Iuliu Hațieganu” University of Medicine and Pharmacy, 400370 Cluj-Napoca, Romania; 3 1st Clinic and Polyclinic of Internal Medicine, Medical Clinic 2, Clinic of Worms, Department of Diabetology and Endocrinology, University Medical Center, 55131 Mainz, Germany; zimmeran@uni-mainz.de; 4 Department of Pharmacology, Toxicology and Clinical Pharmacology, “Iuliu Hațieganu” University of Medicine and Pharmacy, 400012 Cluj-Napoca, Romania; stefanvesa@gmail.com; 5 Emergency Pediatric Hospital, 400370 Cluj-Napoca, Romania; ioana_nascu1@yahoo.com (I.N.); victoria_cret@yahoo.com (V.C.); mirelacrisan_cluj@yahoo.co.uk (M.C.); carmen.asavoaie@gmail.com (C.A.); 6 Department of Medical Genetics, “Iuliu Hațieganu” University of Medicine and Pharmacy, Emergency Pediatric Hospital, 400012 Cluj-Napoca, Romania; bolca12diana@yahoo.com; 7 Faculty of Medicine, “Iuliu Hațieganu” University of Medicine and Pharmacy, 400012 Cluj-Napoca, Romania; p_grigorescusido@yahoo.com

**Keywords:** Gaucher disease, pulmonary hypertension, valvular heart disease

## Abstract

Gaucher disease (GD), one of the most common lysosomal disorders, is characterised by clinical heterogeneity. Cardiac involvement is rare and refers to pulmonary hypertension (PH), valvular abnormalities and myocardial infiltrative damage. The aim of this study was to evaluate cardiac involvement in a group of Romanian GD patients. Phenotypic and genotypic characterisation was carried out in 69 patients with GD type 1. Annual echocardiography and electrocardiography were performed to assess pulmonary pressure, morphology and function of the valves and electrocardiographic changes. Nine patients (13%) exhibited baseline echocardiographic signs suggesting PH. Mitral regurgitation was present in 33 patients (48%) and aortic regurgitation in 11 patients (16%). One patient presented aortic stenosis. Significant valvular dysfunction was diagnosed in 10% of patients. PH was associated with greater age (*p* < 0.001), longer time since splenectomy (*p* = 0.045) and longer time between clinical onset and the start of enzyme replacing therapy (*p* < 0.001). Electrocardiographic changes were present in five patients (7%).

## 1. Introduction

Gaucher disease (GD), one of the most common lysosomal storage disorders, is characterised by vast clinical heterogeneity consisting of organomegaly, hematologic problems and skeletal involvement. It is autosomal recessively inherited and is caused by mutations in the gene encoding lysosomal glucocerebrosidase (GBA) resulting in glucosylceramide accumulation, predominantly within splenic, hepatic, bone marrow and pulmonary tissues [1,2,3,4,5]. The gene for GBA encompasses 11 exons and is located on chromosome 1q21, near to a highly homologous pseudogene sequence. To date, more than 400 different mutations have been identified. Very rarely, GD can also be caused by a deficiency in saposin C, a GBA activator [5,6]. The disorder is panethnic, and its prevalence varies between 1/40,000 and 1/100,000 individuals, rising to 1/800 in Ashkenazi Jews [2,3,4]. The genotype–phenotype correlations are very complex, with considerable clinical heterogeneity within the same genotype [7]. The severity of this disease varies widely; some patients are asymptomatic, and others present virtually all possible complications.

Classically, the disorder was classified into three distinct types, delineated by the absence or presence of neurologic involvement and its progression. Type 1, non-neuronopathic Gaucher disease, is the most common subtype and is associated with varying degrees of hepatosplenomegaly, anaemia, thrombocytopaenia, various skeletal complications and, in a small number of cases, lung involvement, which can lead to pulmonary hypertension (PH) and hepatopulmonary syndrome [1,2,3,4,5,8]. Type 2, acute neuronopathic Gaucher disease, is a rapidly progressive neurodegenerative disorder resulting in death within the first years of life. Type 3, chronic neuronopathic Gaucher disease, is a progressive, chronically neurodegenerative form that is present between infancy and adolescence [2,4,5]. A rare form of GD (type 3c) has also been described, consisting of oculomotor apraxia, calcifications of the mitral and aortic valves, and corneal opacities [9,10].

Cardiac involvement is rare and refers to PH, calcification of the valves and aorta, decreased cardiac output and left ventricle diastolic dysfunction in the context of myocardial infiltrative damage, restrictive cardiomyopathy, left ventricular hypertrophy, pericardial calcification and constrictive pericarditis [7,9,10,11,12,13,14,15,16,17,18,19,20]. Although many studies concerning clinical manifestations in GD have been published, few of these studies investigated cardiac involvement in this disorder in large groups of patients. Of these few studies, most have been published in Israel, where the increased incidence of the disease is well known and have involved echocardiographic and cardiac magnetic resonance assessment of the heart [7,11]. Many other studies are individual case reports and clinical observations [10,12,13,14,15,16,17,18,19,20,21]. The factors that predispose patients with GD to PH are not well understood. Mistry et al. demonstrated a correlation between PH and genetic factors such as non-N409S *GBA* mutation, angiotensin converting enzyme (ACE) gene polymorphism and epigenetic modifiers (asplenia) in GD type 1 patients [11]. Valvular involvement was predominantly described in GD type 3 homozygous for the D409H mutation and consisted of calcification of the valves [10,12,13]. We hypothesised that there are other epigenetic factors contributing to PH in GD patients. Based on previous reported data, the aim of our study was to evaluate cardiac involvement in a group of Romanian GD type 1 patients, including evaluating PH and valvular involvement, to assess a number of factors (age at clinical onset, age at treatment initiation, duration between clinical onset and treatment initiation, enzyme replacing therapy duration, splenectomy, post-splenectomy duration, severity score, valvular abnormalities and genotype) in relation to the occurrence of PH and to find a genotype–phenotype correlation in patients with valvular impairment.

## 2. Materials and Methods

### 2.1. Study Design

This was a monocentric, retrospective, observational, analytical cohort study.

### 2.2. Study Population

From a group of 77 patients diagnosed in our centre, representing all Romanian GD patients, 69 patients met the criteria for inclusion in this study. We performed full phenotypic/genotypic characterisation for these 69 patients (28 males, 41 females; six children and 63 adults; mean age 42 ± 15 years; range 7–75 years), whom we followed at the National Centre for Lysosomal Diseases at Emergency Pediatric Hospital Cluj-Napoca, Romania during the period 2014–2018. Depending on age, the patients were divided into four groups: ≤18-years-old (six patients); >18-years-old and ≤40-years-old (26 patients); >40-years-old and ≤60-years-old (28 patients); and >60-years-old and ≤80-years-old (nine patients).

### 2.3. Methods

The following data were registered: age at diagnosis, age at starting of enzyme replacement therapy (ERT), actual age, duration of ERT, medical history of cardiac disease, genotype, and splenic status. Regular annual follow-up was performed according to the international recommendations, including echocardiography and ECG, and all data were collected during this regular annual follow-up. Twenty-four patients were asplenic, with the splenectomies having been performed prior to ERT. Patients had a confirmed diagnosis of GD based on low leukocyte acid β-glucosidase activity supplemented by *GBA* genotyping. All patients were undergoing ERT with human recombinant GBA (imiglucerase, Cerezyme), intravenous infusion every two weeks, 30–60 U/kg, according to disease severity.

Severity of disease was estimated according to the disease severity scoring system [22].

Transthoracic echocardiography (TTE) was performed by the same physician for all patients, in standard fashion, using a commercially available system (Vivid S6, General Electric, Wauwatosa, WI, USA) with a transducer, according to the age of patient. All cardiac measurements were conducted in accordance with the recommendations of the European Association of Cardiovascular Imaging for cardiac chamber and PH quantification [23,24]. The standardised protocol included scanning in the parasternal long axis, parasternal short axis, apical and subcostal views.

For each patient, using two-dimensional guided M-mode, the following parameters were recorded: end-diastolic left-ventricular internal dimension, inter-ventricular septum thickness in diastole, left-ventricular posterior wall thickness in diastole, ejection fraction and shortening fraction. Tricuspid regurgitation peak velocity (TRV) was measured using continuous wave Doppler. Diastolic filling was established using the E/A ratio by measuring mitral-inflow as determined by pattern-peak early filling (E) and late filling (A) velocities. An E/A ratio < 1 was considered abnormal. Morphology and thickness of the valves was carefully evaluated, and valve regurgitation was determined according to the international recommendations [24]. Measurements were averaged over five cardiac cycles in atrial fibrillation. As echocardiography can only estimate the probability of PH, we used TRV with other echocardiographic markers, including pulmonary artery (PA) diameter, early diastolic pulmonary regurgitation velocity (PRV), eccentricity index (EI), right atrial area, inferior vena cava (IVC) diameter, presence of pericardial effusion, right ventricle (RV) dimensions, fractional area change (FAC), tricuspid annular plane systolic excursion (TAPSE) and pulmonary acceleration time (PAT). In adults, PA diameter > 25 mm was considered abnormal [25]. RV dimensions included basal diameter (dimension > 41 mm was considered abnormal in adults) and the length of the RV (dimension > 83 mm was considered abnormal in adults) [23]. In children, Z score was calculated using www.parameterz.com (accessed on 10 September 2020). EI represented the ratio between the left ventricular diameter perpendicular to the septum and left ventricular diameter parallel to the septum measured during end-systole, and a value > 1.1 was considered abnormal (25). FAC was calculated using the formula FAC = (RVAd − RVAs)/RVAd, where RVAd is RV aria in diastole and RVAs is RV area in systole. FAC < 35% indicates RV systolic dysfunction (23), TAPSE < 1.7 cm was considered suggestive of RV systolic dysfunction (23) and PAT < 100 ms was considered abnormal [26,27].

We also calculated systolic pulmonary artery pressure (SPAP) and mean pulmonary artery pressure (PAP). SPAP was calculated using a modified Bernoulli equation based on the highest TRV (SPAP = 4V^2^ + estimated right arterial pressure). TRV was measured using only signals that were complete (incomplete signals or those contaminated by artefacts were excluded), and a value < 2.8 m/s was considered normal. Right atrial pressure (RAP) was estimated based on IVC size and response to sniff, as recommended by American Society of Echocardiography guidelines (IVC size < 2.1 cm and >50% decrease with sniff = 3 mmHg; IVC < 2.1 cm and >50% decrease with sniff = 8 mmHg; IVC > 2.1 cm with >50% decrease with sniff = 8 mmHg; and IVC > 2.1 cm and <50% decrease with sniff = 15 mmHg) [28]. Mean PAP was calculated using the formula: mean PAP = 4PRV^2^ + estimated RAP [29,30,31].

Significant valvulopathy was considered when the grade of insufficiency or stenosis was ≥2 (moderate or severe).

*Twelve-channel electrocardiogram* recording was performed using the device BTL-08 MT Plus, UK.

### 2.4. Statistical Analysis

Statistical analyses were performed using the MedCalc^®^ Statistical Software version 19.6 (MedCalc Software Ltd., Ostend, Belgium; https://www.medcalc.org; (accessed on 18 October 2020). Normality of distribution for continuous variables was tested using the Shapiro–Wilk test. Continuous variables were characterised by mean and standard deviation (normal distribution) or median and 25–75 percentiles (non-normal distribution). Nominal/categorical variables were expressed as frequency and percentage. Comparisons between two groups (PH vs. without PH patients; asplenic patients vs. without splenectomy) of continuous variables that followed a non-normal distribution (age, age at clinical onset, SPAP, post-splenectomy duration, ERT duration, period between the clinical onset and treatment initiation, age at treatment initiation, mean PAP, TAPSE, PAT, EI, RA, FAC, severity score) were performed using the Mann–Whitney test. Comparisons among more than two groups (age categories) of continuous variables that followed a non-normal distribution (age at clinical onset, post-splenectomy duration, ERT duration, period between the clinical onset and treatment initiation, severity score) were performed using the Kruskal–Wallis test. Comparisons of nominal/categorical variables between groups were performed using the chi-square test (gender, splenectomy, genotype N409S), Fisher test (for small samples in a contingency Table 2 × 2) or the Freeman–Halton extension of the Fisher’s exact probability test (for small samples in a contingency table larger than 2 × 2). Statistical significance was established by calculating the *p* value, with a statistical significance threshold of 0.05 [32].

### 2.5. Ethics Statement

Written informed consent was obtained from adult patients and from guardians of paediatric patients, and the study followed the ethical guidelines of the Declaration of Helsinki and the ethical standards of the national research committee. The study protocol was approved by the Medical Ethics Committee of the University of Medicine and Pharmacy Cluj-Napoca, Romania (no. 353/8.10.2014).

## 3. Results

### 3.1. Baseline Characteristics of Patients with Gaucher Disease

Baseline characteristics of patients with Gaucher disease are depicted in Table 1.

**Figure 1:**
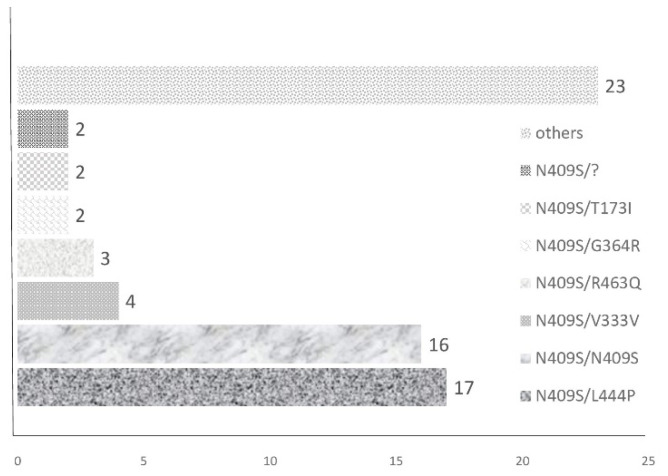
Genotype of GD type 1 patients. Others: N409S/recA456P, N409S/RecNcil, N409S/c.398*; G394R, N409S/c.115 + 1G>T, N409S/1265_1319del, N409S/D419N, N409S/D438N, N409S/F252S, N409S/G416H, N409S/G429V, N409S/H350, N409S/R159W, N409S/R463C, N409S/R463H, N409S/S146L, N409S/T270I, N409S/T291I, N309S/H350P, N309S/RecNcil, N409S/R502H, R463H/R463H, W223R/N409S, p[W387*]; [W387*].

Among the patients included in this study, splenectomy had been performed for cytopaenia or pressure symptoms at the median age of 17 years (range 2–43 years; Table 1). Elevated arterial pressure was diagnosed in 10 patients (14%) and *GBA* genotyping identified the N409S mutation on one or both alleles in 63 patients (91%; Figure 1). No patients possessed the D409H allele.

### 3.2. Pulmonary Hypertension

**Figure 2:**
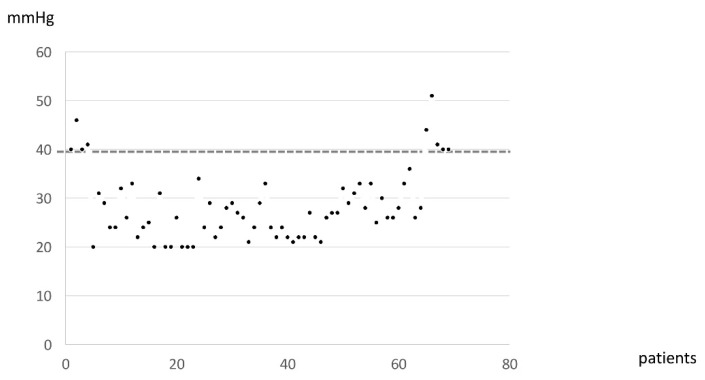
The distribution of SPAP estimated by Doppler echocardiography in GD patients.

Systolic pulmonary artery pressure (SPAP) was estimated by cardiac ultrasound. Most patients exhibited an SPAP under 30 mmHg, with the mean SPAP being 28 mmHg (Figure 2).

Nine patients (13%; median age 66 years, range 53–75 years) presented baseline echocardiographic signs suggesting PH. Five of these patients had undergone splenectomy (median age 64 years, range 59–75 years) and the other four had an intact spleen (median age 69 years, range 63–74 years; *p* = 0.1209). The mean SPAP in this group was 41 mmHg. The characteristics of GD patients with PH are summarised in Table 2.

Patients with PH appear to have some additional distinctive clinical characteristics compared those without PH (Table 3). PH was diagnosed in older patients (*p* < 0.001), with a longer period of time between clinical onset and treatment initiation (*p* < 0.001), a longer period of time since splenectomy (*p* = 0.045) and valvulopathy (*p* < 0.001). Patients with PH appeared to have received ERT for a shorter period of time than had those without PH, but the difference was not significant (*p* = 0.232). No relation was found between genotype, age, treatment initiation, severity score and the presence of PH.

### 3.3. Cardiac Involvement in GD Depending on Age

Clinical parameters and heart involvement were also evaluated for each age group to better assess the evolution of cardiac disease (Table 4).

### 3.4. Cardiac Involvement Depending on the Splenic Status

The characteristics of asplenic patients compared with those without splenectomy are presented in Table 5.

There was a trend toward more asplenic patients having PH (21% with PH vs. 9% without), but this was not significant (*p* = 0.160) due to the small group of patients. In GD patients with splenectomy, there was a significant trend towards higher median age (*p* = 0.016) and higher SPAP (*p* = 0.025). A greater number of asplenic patients had experienced valvular abnormalities compared to those without splenectomy (62% vs. 44%), but the difference was not significant (*p* = 0.053).

### 3.5. Valvular Disease

**Figure 3:**
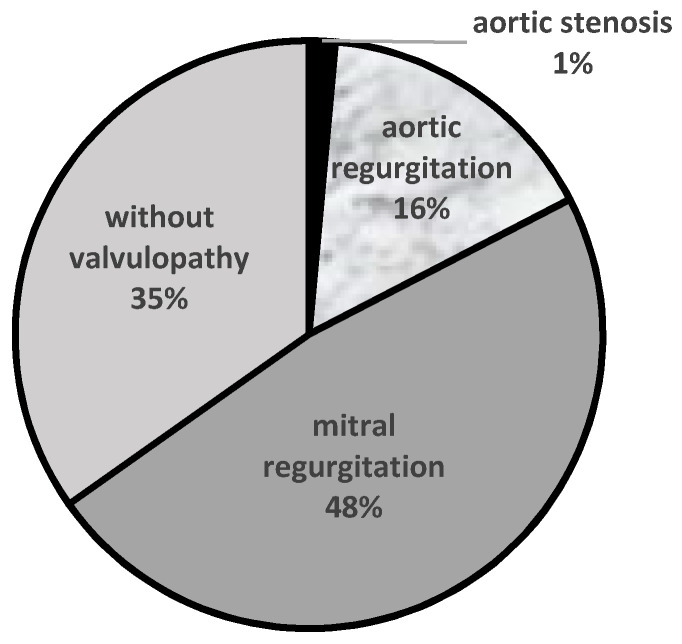
Valvular disease in GD patients.

Among the 69 patients with GD participating in this study, 35 patients (51%) exhibited valvular abnormalities. Mitral regurgitation of various grades was present in a total of 33 patients (48%)–28 (41%) with mild regurgitation, four (6%) with moderate regurgitation and one (1%) with severe mitral regurgitation. In contrast, aortic regurgitation was found in 11 patients (16%)–seven (10%) with mild aortic regurgitation and four (6%) with moderate aortic regurgitation. One patient presented with mild aortic stenosis (1%), and nine patients exhibited both mitral and aortic valvulopathy. Significant (moderate or severe) mitral and/or aortic regurgitation was present in 10% of patients. All patients with PH exhibited aortic and/or mitral regurgitation (Figure 3), and PH was diagnosed in 26% of patients with valvular dysfunction. None of the GD patients from our group presented with valvular calcifications.

Genotypes of the patients with valvular involvement are presented in Table 6. We found no correlation between the most common mutations and the presence of valvular involvement or its severity.

### 3.6. Electrocardiographic Changes

Electrocardiographic changes in our group were present in five patients (7%). These changes consisted of left ventricle hypertrophy (two patients; one with dilative cardiomyopathy, one with aortic regurgitation and hypertension), left atrial enlargement (two patients; one with severe mitral regurgitation, one with dilative cardiomyopathy), right atrial hypertrophy (two patients; one with moderate mitral regurgitation, one with dilative cardiomyopathy), atrial fibrillation (two patients; one with dilative cardiomyopathy, one with moderate mitral regurgitation), ventricular extrasystoles (two patients with structurally normal hearts) and first-degree atrioventricular block (one patient with moderate mitral regurgitation). Four of these five patients exhibited more than two electrocardiographic changes each.

## 4. Discussion

Gaucher disease is a multisystemic disorder with various acute and chronic complications [33]. Cardiac involvement in GD is thought to be rare. A few studies have investigated cardiac calcification, cardiomyopathy, left ventricular hypertrophy and pericardial disease, but studies estimating the incidence of PH in GD patients are scarce and most of them refer to clinical case reports [7,9,10,11,12,13,14,15,16,17,18,19,20]. Studies on a larger number of patients (range of 71–134 patients) have been published in Israel where the higher incidence of GD is well known (two studies) [7,34] and USA and Canada [11]. Our study involved a small group of patients, representing most of the patients diagnosed in our country and a significant number of possible patients (estimating 190 patients for a population of 19,000,000 inhabitants), but given the variable severity of this disease, many patients carrying the homoallelic N409S mutation are asymptomatic or can have mild manifestations and therefore are not discovered because the disease is rare and very often is not included in the usual list of differential diagnoses [2].

### 4.1. Pulmonary Hypertension

PH is defined by a mean PAP > 25 mmHg at rest [25]. The diagnostic method of choice for PH is heart catheterisation, but in clinical practice transthoracic Doppler echocardiography is used for screening and monitoring PH because it is non-invasive and has good sensitivity, although some studies have reported over- and underestimation of PH by echocardiography [25,35,36,37]. SPAP is the most used parameter for PH assessment in echocardiography. Because information obtained from echocardiography can only grade the probability of PH, the European Society of Cardiology recommends the inclusion of “additional echocardiographic variables suggestive of PH” in the echocardiographic assessment of PH [25]. In this study, we used eight different variables for a better assessment: pulmonary artery diameter, early diastolic pulmonary regurgitation velocity, eccentricity index, right atrial area, right ventricle dimensions, fractional area change, tricuspid annular plane systolic excursion and pulmonary acceleration time [25,26,27,28,29,30,31].

Variable incidence of elevation of PAP has recently been reported in patients with GD, varying between 7.4% in ERT patients and 30% in non-treated patients [7,11,38,39,40]. Data originating from the International Gaucher Collaborative Group showed that mild PH was diagnosed in 13% of patients [2]. Despite PH affecting a minority of GD patients, it can represent a life-threatening disease that, before the ERT era, was recognised as a cause of premature mortality [41].

Nine patients in our group (13%) exhibited a mild form of PH. All nine were adults and all had associated valvular dysfunction of varying severities. Data concerning the prevalence of PH in the general population are scarce [42] and vary according to age and associated diseases. Previous studies have reported PH in 2.6% of the population in the Netherlands, 6.8% in USA and 8% in Italy [43,44,45]. When additional echocardiographic variables suggestive for PH were used in the assessment of PH, the prevalence raised to 5.4% in the Netherlands and 9.1% in Australia [43,46]. In comparison to the general population, the prevalence of PH in our group was considerably higher. According to age groups, the prevalence of PH was 7% in patients between the ages of 40 and 60 years, and 78% in patients over 60 years of age.

Heart valvular disease could be a factor contributing to the occurrence of PH in our group, but the small number of patients and the increased age of these patients (median age 70 years), which is associated with PH in the general population, cannot fully support this result. Eight patients with PH had two valves affected (mitral and aortic valve) and seven patients had moderate or severe valvular disease.

We found PH in 26% of patients with GD and valvular abnormalities, but this value does not exceed the prevalence of PH in patients with valvular heart disease in the general population, which varies considerably among studies (15–36.7% in aortic stenosis, 25–36.7% in aortic regurgitation and 20–62% in mitral regurgitation) [47,48,49,50,51].

In line with the general literature on PH, our findings could enable identification of GD type 1 patients who are at high risk of developing PH: older patients with splenectomy performed long ago, with a long interval between clinical onset of the disease and the start of ERT. The small number of patients in our study, however, makes this association difficult to support.

Removal of the spleen, which is the primary reservoir of storage cells, will determine the migration of mononuclear phagocytes to other tissues considered to be macrophage pools, such as lung tissue. Other mechanisms that could explain the association of splenectomy and PH are interactions among the dysfunctional pulmonary endothelium, intimal fibrosis, medial hypertrophy, plexiform lesions and thrombocytosis, which appears after splenectomy and the activation of platelets by damaged circulating red cells with expression of anionic phospholipids on their outer leaflets, leading to in situ thrombosis by acting as cofactors for proteolytic reaction [11,25,52,53,54,55]. Over the two decades since the introduction of ERT, the need for splenectomy to control the signs and symptoms of GD, including severe cytopaenia and abdominal discomfort, has been substantially reduced [56,57].

Studies of GD have demonstrated that other genetic or environmental factors likely mediate the clinical expression of the disease, and several mechanisms for this have been proposed, including increased extralysosomal accumulation of glucosylceramides, the presence of megakaryocytes in the lungs due to extramedullary haematopoiesis and plexogenic arteriopathy [58,59,60,61]. Another contribution to pulmonary involvement in GD is related to infiltration of the lungs by Gaucher cells, creating an interstitial disease that can lead to pulmonary fibrosis, occlusion of the vasculature by fat emboli and increased lung surfactant phosphatidylcholine [5,41,62].

Another factor related to PH in our group of GD patients was a longer period of time between clinical onset of the disease and starting therapy, likely because it has been demonstrated in large groups of patients that lung involvement in GD may be prevented or improved by ERT [62,63,64,65]. Higher doses of ERT can improve the therapeutic outcome because the lung represents a sequestered site not readily accessible to ERT due to high uptake in the liver [60].

Regarding the genotype of patients with PH, one patient possessed a nonN409S genotype (R463H/R463H), one patient was homozygous for N409S mutation and seven patients were N409S/heteroallelic (N409S/L444P in two patients and N409S/T173I, N409S/G364R, N409S/R463C, N409S/G429V, N409S/D438N in each of the other patients, respectively), but no significant differences were found related to patients without PH. The common mutant allele N409S is considered a low-severity mutation and neuroprotective, but it does not predict the severity of bone and visceral involvement, and there is an extraordinary heterogeneity in disease severity [1,2,66,67]. Some studies suggest that GD patients homoallelic for the L444P mutation have a major risk of developing intrinsic pulmonary disease at an early age, but this mutation was not detected in our group [68].

Genotype–phenotype correlation in GD is not well defined. Siblings can have the same genotype and different disease manifestations, organ involvement and response to ERT, and, conversely, many patients have unique or very similar disease manifestations and different genotypes, suggesting the influence of other epigenetic/environmental factors [2,59].

PH did not correlate with severity score, suggesting that it depends more on the progression of disease and late initiation of therapy.

### 4.2. Valvular Disease

Valvular disease in our group was represented by mild mitral and aortic regurgitation in most of the patients, although significant mitral and/or aortic regurgitation was present in 10% of patients. The incidence of valvular disease in the general population depends on age (from 0.7% in subjects 18–44 years to 13.3% in subjects 75 years and older) and world region (7.1–13.2% in Eastern Europe) [69,70]. In our group, significant valvular regurgitation was found in patients older than 40 years (4% in group > 40 years to ≤60 years and 67% in group > 60 years). Significant valvular dysfunction found in older patients represented a contributing factor to the development of PH in our group, but this finding cannot be attributed exclusively to GD because aging and valvular disease are associated with PH in the general population, and the low number of patients in our group cannot lead to an undeniable statement. In asplenic patients, greater age also represented a confounder for valvular abnormalities.

We attempted to find a correlation between presence and severity of the valvulopathy and the genotype of patients. The most frequent mutations in Romanian GD type 1 patients comprised the N409S mutation, either in homozygous state or associated with L444P or another allele (in 91% of cases). No significant association was found with valvular involvement, however, suggesting that other genetic, environmental or developmental factors may contribute to the clinical expression of GD.

None of our patients had valvular calcification or homozygous missense variant D409H in the *GBA* gene, which is associated with calcification of the cardiac valves, so it is likely that other genetic or environmental factors mediate the clinical expression of disease. One patient in our group with genotype N409S/R502H exhibited mitral valve prolapse. Studies reporting cardiac involvement in GD are scarce, however, and most of them are reports of isolated cases (Table 7) [7,10,11,12,13,71,72,73,74,75,76,77,78,79,80,81,82,83,84,85,86,87,88,89,90].

### 4.3. Electrocardiographic Changes

Electrocardiographic changes in our group of GD patients consisted of enlargement of the heart chamber consecutively to valvular regurgitation or stenosis and arrhythmia (atrial fibrillation, ventricular extrasystoles and first-degree atrioventricular block). Conduction system abnormalities are related to abnormal accumulation of glucosylceramides in the lysosomes of conduction tissues, and hypertrophy and eventual fibrosis provide a substrate for atrial and ventricular arrhythmia.

In our small group of paediatric patients (six subjects), all undergoing ERT, no cardiac structural or functional abnormalities were found. Other studies of children with GD did not find abnormal echocardiographic examinations either, concluding that children with GD undergoing ERT are apparently not at risk for developing PH [33,91].

Our study has several limitations. First, the small number of patients reported may limit generalisation of the results. Moreover, the group exhibited great variability in age, age at starting ERT and time elapsed since splenectomy. We had a small number of older patients (>60 years) in which the frequency of valvular diseases and PH was higher when compared to younger patients, and this makes the association between different parameters difficult to interpret.

## 5. Conclusions

The results of this study suggest that older patients who had undergone splenectomy a long time ago, with a long period of time between clinical onset and starting ERT, are considered at risk to have PH, with reservations regarding the small number of patients. We found a higher incidence of PH in comparison to other studies, but in line with results reported by the International Gaucher Collaborative Group. The presence of valvular disease in our group was not correlated with mutations in the *GBA* gene, suggesting that other environmental or developmental factors may contribute to the clinical expression of the disease. Further studies are needed to corroborate these findings and to determine all variables influencing PH in GD patients.

## Figures and Tables

**Table 1:** Characteristics of patients with Gaucher disease.

Parameter	N/%	Male	Female	Median Age at Onset (Years)	Median Actual Age (Years)	Median Time from Splenectomy (Years)	Median Duration of ERT (Years)
Asplenic	24/35%	8	16	15	48	29	7
Without splenectomy	45/65%	20	25	21	38	-	7

ERT: enzyme replacing therapy.

**Table 2:** Clinical characteristics and cardiac findings of patients with Gaucher disease type 1 and PH.

Patient ID	Sex	Age (Years)	Age at Clinical Onset (Years)	Age at Diagnosis (Years)	Treatment Duration (Years)	Duration from Splenectomy (Years)	*GBA* Genotype	Echocardiographic and ECG Findings
1	M	63	42	59	4	-	N409S/ L444P	MR grade II Dilative cardiomyopathy Left ventricle enlargement Left atrial enlargement HTN Atrial fibrillation
2	F	71	35	64	4	-	R463H/R463H	MR grade III AR grade I Left atrial enlargement Right atrial enlargement Atrial fibrillation
3	M	74	12	71	4	-	N409S /L444P	MR grade I AR grade II Mild AS HTN
4	M	67	15	61	6	-	N409S/ T173I	MR grade I AR grade II
5	F	53	4	48	4	43	N409S/ G364R	MR grade II AR grade I TR grade II Right atrial enlargement First degree AV block
6	M	75	64	70	4	10	N409S/ R463C	MR grade II AR grade II Left ventricle hypertrophy Left atrial enlargement HTN
7	M	59	4	45	14	48	N409S /G429V	MR grade I AR grade I
8	F	63	19	58	4	42	N409S/ D438N	MR grade I AR grade I HTN
9	F	70	35	64	4	31	N409S/ N409S	MR grade I AR grade II

AR: aortic regurgitation; AV: atrioventricular; HTN: hypertension; MR: mitral regurgitation; TR: tricuspid regurgitation.

**Table 3:** Characteristics of GD patients with PH compared to those without PH.

Parameter		PH (9 Patients/13%)	Without PH (60 Patients/87%)	*p*
Age (years) (median; 25–75 percentiles)		70 (63; 74)	38 (32; 47)	<0.001 *
Age at clinical onset (years) (median; 25–75 percentiles)		14 (6; 29)	35 (15; 45)	0.167 *
Gender F: M		4:5	37:23	0.433 **
SPAP mmHg (median; 25–75 percentiles)		41 (38; 45)	26 (22; 29)	<0.001 *
% splenectomy (n/%)		5/56%	19/32%	0.259 **
Post-splenectomy duration (years) (median; 25–75 percentiles)		42 (31; 48)	21 (12; 38)	0.045 *
Genotype N409S *GBA* allele (n/%)		8/89%	55/92%	0.514 **
ERT duration (years) (median; 25–75 percentiles)		4 (4; 4)	8 (5; 12)	0.232 *
Period between the clinical onset and treatment initiation (years) (median; 25–75 percentiles)		31 (17; 46)	12 (3; 19)	<0.001 *
Age at treatment initiation (years) (median; 25–75 percentiles)		53 (16; 63)	30 (17; 46)	0.090 *
Presence of valvulopathy	Aortic and mitral valvulopathy (n/%)	8/89%	1/2%	<0.001 ***
	Presence of aortic valvulopathy (n/%)	8/89%	2/3%	<0.001 ***
	Presence of mitral valvulopathy (n/%)	9/100%	24/40%	<0.001 **
	Severity of valvulopathy (≥ grade II) (n/%)	9/100%	0/0%	<0.001 ***
Severity score (median and 25–75 percentiles)		12 (8; 13)	13 (10; 14)	0.338 *

ERT: enzyme replacement therapy; GBA: glucocerebrosidase; PH: pulmonary hypertension; SPAP: systolic pulmonary artery pressure; * Mann–Whitney test; ** chi-square test; *** Fisher’s exact test.

**Table 4:** Clinical characteristics and cardiac involvement of Gaucher patients depending on age.

Parameter		Age Group				*p*
		≤18 Years-Old (6 Patients /9%)	>18 Years-Old ≤ 40 Years-Old (26 Patients/38%)	>40 Years-Old ≤ 60 Years-Old (28 Patients/40%)	>60 Years-Old ≤ 80 Years-Old (9 Patients/13%)	
Age at onset GD (years) (median; 25–75 percentiles)		5 (3;8)	13 (8;21)	21 (5; 34)	19 (12; 3)	0.019 *
Splenectomy (n/%)		0%	7/27%	13/46%	4/44%	0.114 **
Years post-splenectomy (median; 25–75 percentiles)		0	21 (14; 22)	37 (23; 45)	36 (15; 45)	0.043 *
Genotype N409S *GBA* allele (n/%)		5/83%	24/92%	26/93%	8/89%	0.879 ***
ERT duration (years) (median; 25–75 percentiles)		8 (4; 11)	9 (3; 12)	6 (4;12)	4 (4; 9)	0.846 *
Period between clinical onset and treatment initiation (years) (median; 25–75 percentiles)		1 (1; 2)	11 (3; 15)	17 (3; 37)	35 (19; 43)	<0.001 *
Valvulopathy (n/%)	Presence of valvulopathy	0/0%	8/31%	19/68%	8/89%	<0.001 **
	Aortic valvulopathy	0/0%	1/4%	3/11%	7/78%	<0.001 **
	Mitral valvulopathy	0/0%	7/27%	18/64%	8/89%	<0.001 **
	Total (n = 35)	0	8	19	8	
Severity of valvulopathy (≥grade II)		0/0%	0/0%	1/4%	7/78%	<0.001 **
Severity score (median; 25–75 percentiles)		10 (7; 12)	12 (9;14)	13 (12;15)	12 (8;12)	0.077 *
Pulmonary hypertension (n/%)		0/0%	0/0%	2/7%	7/78%	<0.001 **

ERT: enzyme replacement therapy; GBA: glucocerebrosidase; * Kruskal–Wallis test; ** Freeman–Halton extension of the Fisher’s exact probability test *** chi-square test.

**Table 5:** Clinical and echocardiographic characteristics of asplenic patients compared with those without splenectomy.

Parameter		Asplenic Patients 24 Patients/35%	Without Splenectomy 45 Patients/65%	*p*
Age years (median; 25–75 percentiles)		47 (27; 59)	40 (32; 48)	0.016 *
Gender F: M		16:8	25:20	0.285 **
Patients with PH (n/%)		5/21%	4/9%	0.160 ***
SPAP (mmHg) (median; 25–75 percentiles)		29 (27; 35)	25 (22; 30)	0.025 *
Mean PAP (mmHg) (median; 25–75 percentiles)		20 (17; 27)	17 (15; 20)	0.301 *
TAPSE (mm) (median; 25–75 percentiles)		22 (20; 25)	22 (20; 25)	0.302 *
PAT (ms) (median; 25–75 percentiles)		151 (127; 183)	148 (126; 174)	0.120 *
EI (median; 25–75 percentiles)		1 (1; 1.2)	1 (1; 1.2)	0.138 *
RV dimensions ^^^ (median; 25–75 percentiles)	basal diameter (mm)	34 (32; 39)	34 (32; 36)	0.052 *
	length (mm)	73 (66; 77)	70 (68; 74)	0.093 *
RA aria (cm^2^) ^^^ (median; 25–75 percentiles)		15 (13; 19)	14 (12; 16)	0.217 *
FAC (%) (median; 25–75 percentiles)		36 (33; 39)	34 (29; 40)	0.221 *
Genotype N409S *GBA* allele (n/%)		23/96%	40/89%	0.329 **
ERT duration (years) (median; 25–75 percentiles)		6 (4; 13)	7 (1.5; 18)	0.202 *
Period between clinical onset and treatment initiation (years) (median; 25–75 percentiles)		25 (13; 38)	12 (3; 20)	0.270 *
Associated valvulopathy (n/%)		15/62%	20/44%	0.053 **
Severity score (median; 25–75 percentiles)		15 (13; 20)	12 (8; 13)	<0.001 *

EI: eccentricity index; FAC: fractional aria change; GBA: glucocerebrosidase; PAP: pulmonary artery pressure; PAT: pulmonary acceleration time; RA: right atrium; RV: right ventricle; SPAP: systolic pulmonary artery pressure; TAPSE: tricuspid annular plane systolic excursion; ^: adult patients; * Mann–Whitney test; ** chi-square test; *** Fisher’s exact test.

**Table 6:** Genotype of patients with valvulopathy.

Genotype	Patients (N)			*p*
	with Valvulopathy (35 Patients)		without Valvulopathy (34 Patiens)	
	Mild (28 Patiens)	Moderate and Severe (7 Patients)		
N409S/N409S	7	1	8	0.9472 *
N409S/L444P	5	2	10	0.3643 *
N409S/other	14	3	14	0.5369 *
Non N409S allele	2	1	2	0.6665 *

* chi-square test.

**Table 7:** Cardiovascular involvement in Gaucher disease.

Study	Country of Origin	Number of Patients	Age (Years)	Cardiovascular Involvement				Genotype
				Valvular	Vascular	Cardiomyopathy	PH	
Abrahamov et al. [12]	Israel	12	2–20	Aortic and mitral valve calcification	-	-	-	D409H/D409H
Aksu et al. [71]	Turkey	1	20	Aortic and mitral valve calcification	-	-	-	NA
Alsahli et al. [72]	Saudi Arabia	1	11	Valvular and aortic calcification	-	-	-	D409H/D409H
Beutler et al. [73]	Great Britain, Germany	1	18	NA	-	-	-	D409H/D409H
Cainelli et al. [74]	Kazakhstan	1	17	NA	-	-	-	N409S/L483P
Casta et al. [75]	USA	1	15	Aortic and mitral valve calcification	Calcification of the ascending aorta	-	-	NA
Chábas et al. [13]	Spain	3	10, 16, 17	Aortic and mitral valve calcification	Calcification of the ascending aorta	-	-	D409H/D409H
Cindik et al. [76]	Turkey	1	14	Aortic and mitral valve stenosis		-	-	D409H/D409H
Cho et al. [77]	Palestine	1	18	Aortic and mitral valve stenosis	Calcification of the aortic root	-	-	D409H/D409H
Elstein et al. [7]	Israel	134	NA	NA	NA	-	7% of ERT patients	N409S allele in all patients with PH
Erdurdan et al. [78]	Turkey	1	12	-	-	LV hypertrophy	-	NA
George et al. [79]	Palestine	1	17	Aortic and mitral valve stenosis	Calcification of the aortic root	-	-	D409H/D409H
Kör et al. [10]	Turkey	1	15	Aortic and mitral valve calcification	Calcification of the ascending aorta	LV hypertrophy	-	D409H/D409H
Kurolap et al. [80]	Israel	3	14 18 15	Mitral and pulmonary valve calcification Calcification of the aortic valve	Calcification of the aorta Calcification of the aorta	Heart failure	-	D409H/D409H
Michelakakis et al. [81]	Greece	1	0.7	Aortic valve insufficiency	-	LH hypertrophy	-	D409H/D409H
Mireles et al. [82]	Mexico	1	10	Aortic and mitral valve calcification	-	-	-	D409H/D409H
Mistry et al. [11]	Canada, USA	40 untreated patients 94 patients on ERT	49 ± 21 43 ± 11	NA	NA	NA	PH in 30% of untreated patients PH in 10% of ERT patients	Non N409S allele in 78% of patients with severe PH
Odrizola et al. [83]	Spain	1	29	-	-	-	+	N409S/D55
Rastogi et al. [84]	India	1	16	Aortic and mitral valve calcification	-	LV hypertrophy	-	N409S/D55
Rosengarten et al. [34]	Israel	71	9.8 ± 5	-	-	-	-	NA
Saraclar et al. [85]	Turkey	2	9, 15	Aortic and mitral valve calcification	-	-	-	NA
Sharratt et al. [86]	Canada	1	12	Aortic and mitral valve calcification	-	Calcification of the left atrium	-	NA
Solanich et al. [21]	Spain	1	62	-	-	Myocardial infiltration	-	N409S/L444P
Stone at al. [87]	USA	1	6	-	Intimal fibrosis of the coronary arteries and aortic root	LH hypertrophy,	-	D409H/RecNciI
Torloni et al. [88]	Brazil	1	23	Dilative cardiomyopathy	-	-	-	NA
Uyama et al. [89]	Japan	3	27, 35, 42	Aortic and mitral valve calcification	Fibrosis of the ascending aorta	-	-	D409H/D409H
Wilson et al. [90]	USA	1	15	Aortic and mitral valve calcification	Calcification of the ascending aorta	-	-	NA

ERT: enzyme replacing therapy; LV: left ventricle; NA: not available; PH: pulmonary hypertension.

## References

[1] Sidransky E. (2012). Gaucher Disease: Insights from a Rare Mendelian Disorder. Discov. Med..

[2] Grabowski G., Zimran A., Ida H. (2015). Gaucher disease types 1 and 3: Phenotypic characterization of large populations from the ICGG Gaucher Registry. Am. J. Hematol..

[3] Mehta A. (2007). Epidemiology and natural history of Gaucher’s disease. Eur. J. Int. Med..

[4] Mehta A., Kuter D.J., Salek S.S., Belmatoug N., Bembi B., Bright J., Vom Dahl S., Deodato F., Di Rocco M., Göker-Alpan O. (2019). Presenting signs and patient co-variables in Gaucher disease: Outcome of the Gaucher Earlier Diagnosis Consensus (GED-C) Delphi initiative. Int. Med. J..

[5] Stirnemann J., Belmatoug N., Camou F., Serratrice C., Froissart R., Caillaud C., Levade T., Astudillo L., Serratrice J., Brassier A. (2017). A Review of Gaucher Disease Pathophysiology, Clinical Presentation and Treatments. Int. J. Mol. Sci..

[6] Vaccaro A.M., Motta M., Tatti M., Scarpa S., Masuelli L., Bhat M., Vanier M.T., Tylki-Szymanska A., Salvioli R. (2010). Saposin C mutations in Gaucher disease patients resulting in lysosomal lipid accumulation, saposin C deficiency, but normal prosaposin processing and sorting. Hum. Mol. Genet..

[7] Elstein D., Klutstein M.W., Lahad A., Abrahamov A., Hadas-Halpern I., Zimran A. (1998). Echocardiographic assessment of pulmonary hypertension in Gaucher’s disease. Lancet.

[8] de Boer G., van Dussen L., van den Toorn L., den Bakker M., Hoek R., Hesselink D., Hollak C., van Hal P. (2016). Lung transplantation in Gaucher disease: A learning lesson in trying to avoid both Scylla and Charybdis. Chest.

[9] Pashmanik-Chor M., Laadan S., Elroy-Stein O., Zimran A., Abrahamov A., Gatt S., Horowitz M. (1996). The glucocerebrosidase D409H mutation in Gaucher disease. Biochem. Mol. Med..

[10] Kör Y., Keskin M., Bașpinar O. (2017). Severe cardiac involvement in gaucher type IIIC: A case report and review of the literature. Cardiol. Young.

[11] Mistry P.K., Sirrs S., Chan A., Pritzker M., Duffy T., Grace M., Meeker D., Goldman M. (2002). Pulmonary hypertension in type 1 Gaucher’s disease: Genetic and epigenetic determinants of phenotype and response to therapy. Mol. Genet. Metab..

[12] Abrahamov A., Elstein D., Gross-Tur V., Farber B., Glaser Y., Hadas-Halpern I., Ronen S., Takakjdi M., Horowitz M., Zimran A. (1995). Gaucher’s disease variant characterised by progressive calcification of heart valves and unique genotype. Lancet.

[13] Chabàs A., Cormand B., Grinberg D., Burguera J.M., Balcells S., Merino J.L., Mate I., Sobrino J.A., González-Duarte R., Vilageliu L. (1995). Unusual expression of Gaucher’s disease: Cardiovascular calcifications in three sibs homozygous for the D409H mutation. J. Med. Genet..

[14] Talluto C.J., Silverman N.H. (2011). Aortic and mitral valve stenosis with regurgitation: Not due to rheumatic heart disease. Echocardiography.

[15] Altunbas G., Ercan S., Inanç I.H., Ozer O., Kervancioqlu S., Davutoqlu V. (2015). Extensive vascular and valvular involvement in Gaucher disease. Asian Cardiovasc. Thorac. Ann..

[16] Roghi A., Poggiali E., Cassinerio E., Petrotti P., Giuditta M., Milazzo A., Quattrocchi G., Capellini M.D. (2017). The role of cardiac magnetic resonance in assessing the cardiac involvement in Gaucher type 1 patients: Morphological and functional evaluations. J. Cardiovasc. Med..

[17] Lo Iudice F., Barbato A., Muscariello R., Di Nardo C., de Stefano F., Sibilio M., Strazzullo P., de Simone G., Galderisi M. (2015). Left ventricular diastolic dysfunction in type I Gaucher disease: An echo Doppler study. Echocardiography.

[18] Tamari I., Motro M., Neufeld H.N. (1983). Unusual pericardial calcification in Gaucher’s disease. Arch. Intern. Med..

[19] Benbassat J., Bassan H., Milwidski H., Sacks M., Groen J.J. (1968). Constrictive pericarditis in Gaucher’s disease. Am. J. Med..

[20] Harvey P.K.P., Jones M.C., Anderson E.G. (1969). Pericardial abnormalities in Gaucher’s disease. Brit. Heart J..

[21] Solanich X., Claver E., Carreras F., Giraldo P., Vidaller A., Aguilar R., Cequier A. (2012). Myocardial infiltration in Gaucher’s disease detected by cardiac MRI. Int. J. Cardiol..

[22] Zimran A., Kay A., Gelbart T., Garver P., Thurston D., Saven A., Beutler E. (1992). Gaucher disease. Clinical, laboratory, radiologic and genetic features of 53 patients. Medicine.

[23] Lang R.M., Badano L.P., Mor-Avi V., Afilalo J., Armstrong A., Ernande L., Flachskampf F.A., Foster E., Goldstein S.A., Kuznetsova T. (2015). Recommendations for cardiac chamber quantification by echocardiography in adults: An update from the American Society of Echocardiography and the European Association of Cardiovascular Imaging. Eur. Heart J. Cardiovasc. Imaging.

[24] Lancellotti P., Moura L., Pierard L.A., Agricola E., Popescu B.A., Tribouilloy C., Hagendorff A., Monin J.L., Badano L., Zamorano J.L. (2010). European Association of Echocardiography recommendations for the assessment of valvular regurgitation. Eur. J. Echocardiogr..

[25] Galie N., Humbert M., Vachiery J.L., Gibbs S., Lang I., Torbicki A., Simonneau G., Peacock A., Vonk Noordegraaf A., Beghetti M. (2016). 2015 ESC/ERS Guidelines for the diagnosis and treatment of pulmonary hypertension: The joint task force for the diagnosis and treatment of pulmonary hypertension of the European Society of Cardiology (ESC) and the European Respiratory Society (ERS): Endorsed by: Association for European Paediatric and Congenital Cardiology (AEPC), International Society for Heart and Lung Transplantation (ISHLT). Eur. Heart J..

[26] Dabestani A., Mahan G., Gardin J.M., Takenaka K., Burn C., Allfie A., Henry W.L. (1987). Evaluation of pulmonary artery pressure and resistance by pulsed Doppler echocardiography. Am. J. Cardiol..

[27] Tossavainen E., Söderberg S., Grönlund C., Gonzalez M., Henein M.Y., Lindqvist P. (2013). Pulmonary acceleration time in identifying pulmonary hypertension patients with raised pulmonary vascular resistance. Eur. Heart J. Cardiovasc. Imaging.

[28] Rudski L.G., Lai W.W., Afilalo J., Hua L., Handschumacher M.D., Chandrasekaran K., Solomon S.D., Louie E.K., Schiller N.B. (2010). Guidelines for the echocardiographic assessment of the right heart in adults: A report from the American Society of Echocardiography endorsed by the European Association of Echocardiography, a registered branch of the European Society of Cardiology, and the Canadian Society of Echocardiography. J. Am. Soc. Echocardiogr..

[29] Bossone E., D’Andrea A., D’Alto M., Citro R., Argiento P., Ferrara F., Cittadini A., Rubenfire M., Naeije R. (2013). Echocardiography in pulmonary arterial hypertension: From diagnosis to prognosis. J. Am. Soc. Echocardiogr..

[30] Chemla D., Castelain V., Humbert M., Hébert J.L., Simonneau G., Lecarpentier Y., Hervé P. (2004). New formula for predicting mean pulmonary artery pressure using systolic pulmonary artery pressure. Chest.

[31] Abbas A.E., Fortuin F.D., Schiller N.B., Appleton C.P., Moreno C.A., Lester S.J. (2003). A simple method for noninvasive estimation of pulmonary vascular resistance. J. Am. Coll. Cardiol..

[32] Peacock J., Peacock P. (2010). Oxford Handbook of Medical Statistics.

[33] Zimran A., Belmatoug N., Bembi B., Deegan P., Elstein D., Fernandez-Sasso D., Giraldo P., Goker-Alpan O., Lau H., Lukina E. (2018). Demographics and patient characteristics of 1209 patients with Gaucher disease: Descriptive analysis from the Gaucher Outcome Survey (GOS). Am. J. Hematol..

[34] Rosengarten D., Abramov A., Nir A., Farber B., Glaser J., Zimran A., Elstein D. (2007). Outcome of ten years’ echocardiographic follow-up in children with Gaucher disease. Eur. J. Pediatr..

[35] Mclaughlin V., Archer S., Badesch D., Barst R., Farber H., Lindner J., Mathier M., McGoon M., Park H., Rosenson R. (2009). ACCF/AHA 2009 expert consensus document on pulmonary hypertension a report of the American College of Cardiology Foundation Task Force on Expert Consensus Documents and the American Heart Association developed in collaboration with the American College of Chest Physicians; American Thoracic Society, Inc.; and the Pulmonary Hypertension Association. J. Am. Coll. Cardiol..

[36] D’Alto M., Romeo E., Argiento P., D’Andrea A., Vanderpool R., Correra A., Bossone E., Sarubbi B., Calabro R., Russo M.G. (2013). Accuracy and precision of echocardiography versus right heart catheterization for the assessment of pulmonary hypertension. Int. J. Cardiol..

[37] Janda S., Shahidi N., Gin K., Swiston J. (2011). Diagnostic accuracy of echocardiography for pulmonary hypertension: A systematic review and meta-analysis. Heart.

[38] Giraldo P., Alfonso P., Irún P., Gort L., Chabás A., Vilageliu L., Grinberg D., Sá Miranda S., Pocovi M. (2012). Mapping the genetic and clinical characteristics of Gaucher disease in the Iberian Peninsula. Orphanet. J. Rare. Dis..

[39] Jaffe D., Flaks-Manov N., Benis A., Gabay H., DiBonaventura M., Rosenbaum H., Joseph A., Bachrach A., Leventer-Roberts M. (2019). Population-based cohort of 500 patients with Gaucher disease in Israel. BMJ Open.

[40] Kerem E., Elstein D., Abrahamov A., Bar Ziv Y., Hadas-Halpenn I., Melzer E., Cahan C., Branski D., Zimran A. (1996). Pulmonary function abnormalities in type I Gaucher disease. Eur. Resp. J..

[41] Theise N.D., Ursell P.C. (1990). Pulmonary hypertension and Gaucher’s disease: Logical association or mere coincidence?. Am. J. Pediatr. Hematol. Oncol..

[42] Hyduk A., Croft J.B., Ayala C., Zheng K., Zheng Z.J., Mensah G.A. (2005). Pulmonary hypertension surveillance—United States, 1980–2002. MMWR Surveill. Summ..

[43] Moreira E., Gall H., Leening M., Lahousse L., Loth D., Krijthe B., Kiefte-de Jong J., Brusselle G., Hofman A., Stricker B. (2015). Prevalence of Pulmonary Hypertension in the General Population: The Rotterdam Study. PLoS ONE.

[44] Choudhary G., Jankowich M., Wu W.-C. (2013). Prevalence and clinical characteristics associated with pulmonary hypertension in African-Americans. PLoS ONE.

[45] D’Andrea A., Naeije R., Grunig E., Caso P., D’Alto M., Palma E.D., Nunziata L., Riegler L., Scarafile R., Cocchia R. (2014). Echocardiography of the Pulmonary Circulation and Right Ventricular Function: Exploring the Physiologic Spectrum in 1480 Normal Subjects. Chest.

[46] Strange G., Playford D., Stewart S., Deague J., Nelson H., Kent A., Gabbay E. (2012). Pulmonary hypertension: Prevalence and mortality in the Armadale echocardiography cohort. Heart.

[47] Magne J., Pibarot P., Sengupta P., Donal E., Rosenhek R., Lancellotti P. (2015). Pulmonary hypertension in valvular disease. A comprehensive review on pathophysiology to therapy from the HAVEC group. JACC Cardiovasc. Imaging.

[48] Silver K., Aurigemma G., Krendel S., Barry N., Ockene I., Alpert J. (1993). Pulmonary artery hypertension in severe artery stenosis: Incidence and mechanism. Am. Heart J..

[49] Naidoo D.P., Mitha A.S., Vythilingum S., Chetty S. (1991). Pulmonary hypertension in aortic regurgitation: Early surgical outcome. Q. J. Med..

[50] Miller W.L., Mahoney D.W., Enriquez-Sarano M. (2014). Quantitative Doppler-echocardiographic imaging and clinical outcomes with left ventricular systolic dysfunction: Independent impact of pulmonary hypertension. Circ. Cardiovasc. Imaging.

[51] Barbieri A., Bursi F., Grigioni F., Tribouilloy C., Avierinos J.F., Michelena H., Rusinaru D., Szymansky C., Russo A., Suri R. (2011). Mitral Regurgitation International Database (MIDA) Investigators. Prognostic and therapeutic implications of pulmonary hypertension complicating degenerative mitral regurgitation due to flail leaflet: A multicenter long-term international study. Eur. Heart J..

[52] Ennezat P.V., Marechaux S., Huerre C., Deklunder G., Asseman P., Jude B., Van Belle E., Mouquet F., Bauters C., Lamblin N. (2008). Exercise does not enhance the prognosticvalue of Doppler echocardiography in patients with left ventricular systolic dysfunction and functional mitral regurgitation at rest. Am. Heart J..

[53] Weinreb N.J., Goldblatt J., Villalobos J., Charrow J., Cole J.A., Kerstenetzky M., vom Dahl S., Hollak C. (2013). Long-term clinical outcomes in type 1 Gaucher disease following 10 years of imiglucerase treatment. J. Inherit. Metab. Dis..

[54] Crary S.E., Buchanan G.R. (2009). Vascular complications after splenectomy for hematologic disorders. Blood.

[55] Peacock A.J. (2005). Pulmonary hypertension after splenectomy: A consequence of loss of the splenic filter or is there something more?. Thorax.

[56] Palkar A., Agrawal A., Verma S., Iftikhar A., Miller E., Talwar A. (2015). Post splenectomy related pulmonary hypertension. World J. Respirol..

[57] Lo S.M., Liu J., Chen F., Pastores G.M., Knowles J., Boxer M., Aleck K., Mistry P.K. (2011). Pulmonary vascular disease in Gaucher disease: Clinical spectrum, determinants of phenotype and long-term outcomes of therapy. J. Inherit. Metab. Dis..

[58] van Dussen L., Biegstraaten M., Dijkgraaf M.G., Hollak C.E. (2014). Modelling Gaucher disease progression: Long-term enzyme replacement therapy reduces the incidence of splenectomy and bone complications. Orphanet. J. Rare Dis..

[59] Mistry P.K., Liu J., Yang M., Nottoli T., McGrath J., Jain D., Zhang K., Keutzer J., Chuang W.-L., Mehal W. (2010). Glucocerebrosidase gene-deficient mouse recapitulates Gaucher disease displaying cellular and molecular dysregulation beyond the macrophage. Proc. Natl. Acad. Sci. USA.

[60] Lo S.M., Stein P., Mullaly S., Nottoli T., McGrath J., Jain D., Zhang K., Keutzer J., Chuang W.-L., Mehal W. (2010). Expanding spectrum of the association between Type 1 Gaucher disease and cancers: A series of patients with up to 3 sequential cancers of multiple types—Correlation with genotype and phenotype. Am. J. Hematol..

[61] Thachil J. (2009). The enigma of pulmonary hypertension after splenectomy—Does the megakaryocyte provide a clue?. QJM.

[62] Buccoliero R., Palmieri S., Ciarleglio G., Collodoro A., De Santi M., Federico A. (2007). Increased Lung surfactant phosphatidylcholine in patients affected by lysosomal storage diseases. J. Inherit. Metab. Dis..

[63] Elstein D., Zimran A. (2009). Review of the safety and efficacy of imiglucerase treatment of Gaucher disease. Biologics.

[64] Mistry P., Belmatoug N., vom Dahl S., Giugliani R. (2015). Understanding the natural history of Gaucher disease. Am. J. Hematol..

[65] Biegstraaten M., Cox T.M., Belmatoug N., Berger M.G., Collin-Histed T., Vom Dahl S., Di Rocco M., Fraga C., Giona F., Giraldo P. (2018). Management goals for type 1 Gaucher disease:An expert consensus document from the European working group on Gaucher disease. Blood Cells Mol. Dis..

[66] Weinreb N.J., Cappellini M., Cox T., Giannini E., Grabowski G., Hwu W.-L., Mankin H., Martins A.M., Sawyer C., vom Dahl S. (2010). A validated disease severity scoring system for adults with type 1 Gaucher disease. Genet. Med..

[67] Mistry P., Germain D.P. (2006). Phenotype variations in Gaucher disease. Rev. Med. Intern..

[68] Santamaria F., Parenti G., Guidi G., Filocamo M., Strisciuglio P., Grillo G., Farina V., Sarnelli P., Rizzolo M.G., Rotondo A. (1998). Pulmonary manifestations of Gaucher disease: An increased risk for L444P homozygotes?. Am. J. Respir. Crit. Care Med..

[69] Nkomo V., Gardin J., Skelton T., Gottdiener J., Scott C., Enriquez-Sarano M. (2006). Burden of valvular heart diseases: A population-based study. Lancet.

[70] Chen J., Li W., Xiang M. (2020). Burden of valvular heart disease, 1990-2017: Results from the Global Burden of Disease Study 2017. J. Glob. Health.

[71] Aksu T., Baysal E., Biyikkoğlu F., Tüfekçioğlu O. (2011). Gaucher’s disease with valvular, myocardial and aortic involvement in a patient with oculomotor apraxia. Anadolu Kardiyol. Derg..

[72] Alsahli S., Bubshait D., Rahbeeni Z., Alfadhel M. (2018). Aortic calcification in Gaucher disease: A case report. Appl. Clin. Genet..

[73] Beutler E., Kattamis C., Sipe J., Lipson M. (1995). 1342 mutation in Gaucher’s disease. Lancet.

[74] Cainelli F., Nurgaliev D., Nurgalyeva K., Ivanova-Razumova T., Bulanin D., Vento S. (2017). Type 1 Gaucher disease with fatal outcome in a 17-year-old girl from Kazakhstan. Isr. Med. Assoc. J..

[75] Casta A., Hayden K., Wolf W.J. (1984). Calcification of the Ascending Aorta and Aortic and Mitral Valves in Gaucher’s Disease. Am. J. Cardiol..

[76] Cindik N., Ozcay F., Süren D., Akkoyun I., Gökdemir M., Varan B., Alehan F., Ozbek N., Tokel K. (2010). Gaucher disease with communicating hydrocephalus and cardiac involvement. Clin. Cardiol..

[77] Cho L., Lytle B.W., Moodie D.S. (2000). Type IIIC Gaucher’s disease. Circulation.

[78] Erduran E., Mocan H., Gedik Y., Kamaci R., Ökten A., Deger O. (1995). Hydrocephalus, corneal opacities, deafness, left ventricle hypertrophy, clinodactyly in an adolescent patient. A new syndrome associated with glucocerebrosidase deficiency. Genet. Couns..

[79] George R., McMahon J., Lytle B., Clark B., Lichtin A. (2001). Severe valvular and aortic arch calcification in a patient with Gaucher’s disease homozygous for the D409H mutation. Clin. Genet..

[80] Kurolap A., Del Toro M., Speigel R., Gutstein A., Shafir G., Cohen I., Barrabès J., Feldman H.B. (2019). Gaucher disease type 3c: New patients with unique presentations and review of the literature. Mol. Genet. Metab..

[81] Michelakakis H., Skardoutsou A., Mathioudakis J., Moraitou M., Dimitriou E., Voudris C., Karpathios T. (2002). Early-onset severe neurological involvement and D409H homozygosity in Gaucher disease: Outcome of enzyme replacement therapy. Blood Cells Mol. Dis..

[82] Mireles S., Seybold J., Williams G. (2010). Undiagnosed type IIIc Gaucher disease in a child with aortic and mitral valve calcification: Perioperative complications after cardiac surgery. J. Cardiothorac. Vasc. Anesth..

[83] Odrizola M., Ferrero O., Jauregui I., Miguel F. (1998). Alglucerase treatment of type I Gaucher disease with pulmonary involvement. Respir. Med..

[84] Rastogi P., Rao S., Kaur J., Malhotra P., Varma S., Das R. (2016). Gaucher’s Disease with Cardiac Valve Calcification and Stenosis: A Rare Presentation due to Homozygous p.D409H Mutation in a North Indian Family. Indian J. Pediatr..

[85] Saraçlar M., Atalay S., Koçak N., Özkutlu S. (1991). Gaucher’s disease with mitral and aortic involvement: Echocardiographic findings. Pediatr. Cardiol..

[86] Sharratt G.P., Price D., Curtis J.A., Cornel G. (1992). Gaucher’s disease with mitral valve calcification. Pediatr. Cardiol..

[87] Stone D., Tayebi N., Coble C., Ginns E., Sidransky E. (2000). Cardiovascular fibrosis, hydrocephalus, ophthalmoplegia, and visceral involvement in an American child with Gaucher disease. J. Med. Genet..

[88] Torloni M.R., Franco K., Sass N. (2002). Gaucher’s disease with myocardial involvement in pregnancy. Sao Paolo Med. J..

[89] Uyama E., Uchino M., Ida H., Eto Y., Owada M. (1997). D409H/D409H genotype in Gaucher-like disease. J. Med. Genet..

[90] Wilson E.R., Barton N.W., Barranger J.A. (1985). Vascular involvement in type 3 neuronopathic Gaucher’s disease. Arch. Pathol. Lab. Med..

[91] Elstein D., Altarescu G., Abrahamov A., Zimran A. (2018). Children with type 1 Gaucher disease: Changing profiles in the 21st century. Blood Cells Mol. Dis..

